# Bis[3-(2-carboxy­ethen­yl)pyridinium-1-acetato]dichloridozinc(II)

**DOI:** 10.1107/S160053680904793X

**Published:** 2009-11-18

**Authors:** Xue-Hui Jing, Wei-Wei Sun, En-Qing Gao

**Affiliations:** a Shanghai Key Laboratory of Green Chemistry and Chemical Processes, Department of Chemistry, East China Normal University, Shanghai 200062, People’s Republic of China

## Abstract

In the title complex, [ZnCl_2_(C_10_H_9_NO_4_)_2_], the Zn^II^ ion lies on a twofold rotation axis and is four-coordinated by two carboxyl­ate O atoms from two 3-(2-carboxy­ethen­yl)pyridinium-1-acetate ligands in a monodentate mode and two Cl atoms in a distorted tetra­hedral geometry. In the crystal structure, inter­molecular O—H⋯O hydrogen bonds link the mol­ecules into a double-chain structure extending parallel to [101].

## Related literature

For general background to hydrogen bonds, see: Beatty (2003 ▶); Liu *et al.* (2008 ▶); Steiner (2002 ▶). For related structures, see: Mao *et al.* (1999 ▶); Sun *et al.* (2009 ▶); Wu *et al.* (2006 ▶); Zhang *et al.* (2002 ▶).
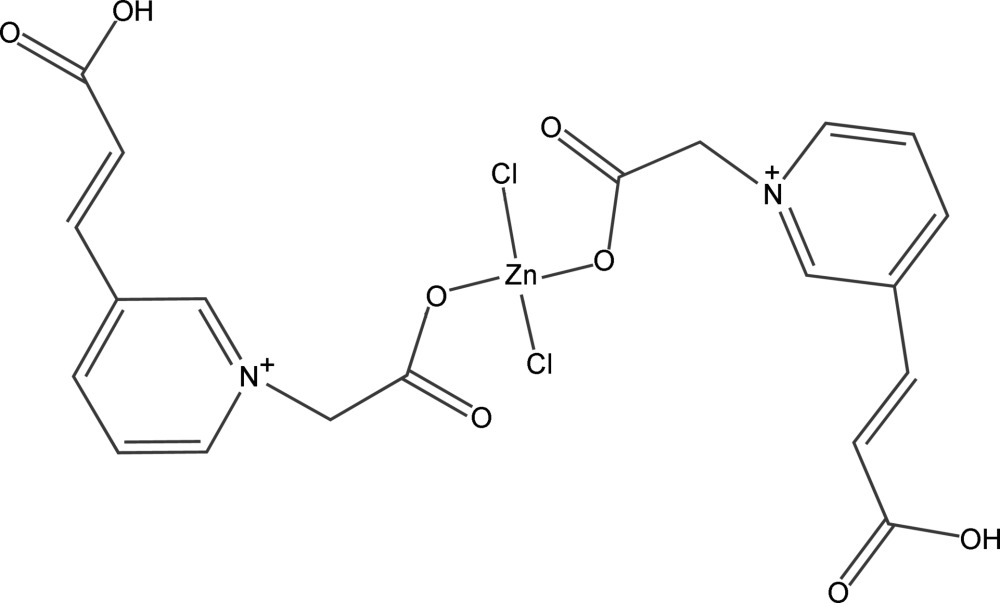


## Experimental

### 

#### Crystal data

[ZnCl_2_(C_10_H_9_NO_4_)_2_]
*M* *_r_* = 550.63
Monoclinic, 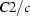

*a* = 16.6247 (6) Å
*b* = 7.1973 (3) Å
*c* = 18.8873 (7) Å
β = 108.685 (1)°
*V* = 2140.81 (14) Å^3^
*Z* = 4
Mo *K*α radiation
μ = 1.45 mm^−1^
*T* = 296 K
0.12 × 0.12 × 0.08 mm

#### Data collection

Bruker APEXII CCD diffractometer
Absorption correction: multi-scan (*SADABS*; Bruker, 2001 ▶) *T*_min_ = 0.845, *T*_max_ = 0.893
27422 measured reflections
2450 independent reflections
2309 reflections with *I* > 2σ(*I*)
*R*_int_ = 0.019

#### Refinement

*R*[*F* ^2^ > 2σ(*F* ^2^)] = 0.028
*wR*(*F* ^2^) = 0.077
*S* = 1.06
2450 reflections
154 parameters
H atoms treated by a mixture of independent and constrained refinement
Δρ_max_ = 0.50 e Å^−3^
Δρ_min_ = −0.24 e Å^−3^

### 

Data collection: *APEX2* (Bruker, 2007 ▶); cell refinement: *SAINT* (Bruker, 2007 ▶); data reduction: *SAINT*; program(s) used to solve structure: *SHELXS97* (Sheldrick, 2008 ▶); program(s) used to refine structure: *SHELXL97* (Sheldrick, 2008 ▶); molecular graphics: *SHELXTL* (Sheldrick, 2008 ▶) and *DIAMOND* (Brandenburg, 1999 ▶); software used to prepare material for publication: *SHELXTL*.

## Supplementary Material

Crystal structure: contains datablocks I, global. DOI: 10.1107/S160053680904793X/hy2249sup1.cif

Structure factors: contains datablocks I. DOI: 10.1107/S160053680904793X/hy2249Isup2.hkl

Additional supplementary materials:  crystallographic information; 3D view; checkCIF report

## Figures and Tables

**Table 1:** Selected bond lengths (Å)

Zn1—O1	1.9983 (13)
Zn1—Cl1	2.2566 (4)

**Table 2:** Hydrogen-bond geometry (Å, °)

*D*—H⋯*A*	*D*—H	H⋯*A*	*D*⋯*A*	*D*—H⋯*A*
O4—H4*B*⋯O2^i^	0.97 (4)	1.60 (4)	2.560 (2)	169 (3)

Symmetry code: (i) 
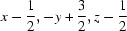
.

**Fig. 1.**
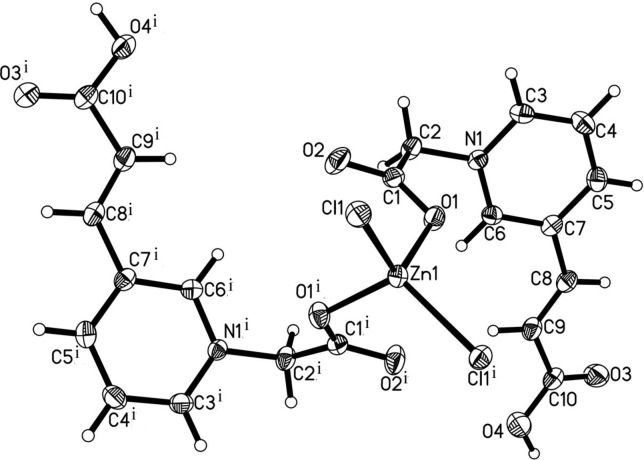
The molecular structure of the title compound. Displacement ellipsoids are drawn at the 30% probability level. [Symmetry code: (i) -x, y, -z + 1/2.]

**Fig. 2.**
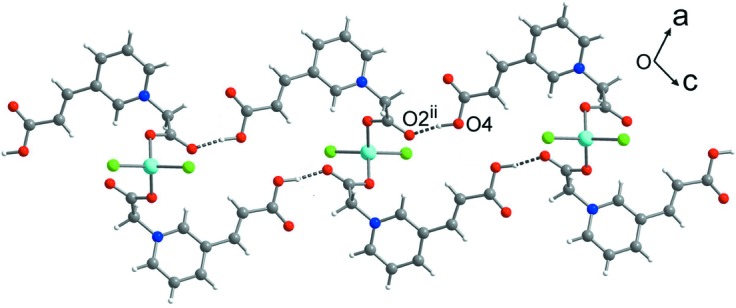
One-dimensional chain structure connected through intermolecular O—H···O hydrogen bonds (dashed lines). [Symmetry code: (ii) x - 1/2, -y + 3/2, z - 1/2.]

## supplementary crystallographic information

### Comment

As we know, hydrogen bond is the most important of all directional intermolecular interactions. It is operative in determining molecular conformation, molecular aggregation, and the function of a vast number of chemical systems ranging from inorganic to biological (Beatty, 2003; Liu *et al.*, 2008; Steiner, 2002). In this paper, we report the coordination and hydrogen-bond structure of the title complex derived from a zwitterionic dicarboxylic ligand, 1-carboxymethylpyridinium-3-acrylic acid. Zwitterionic dicarboxylic acids as ligands have received less attention in coordination chemistry than the common dicarboxylic acids, although an increasing number of coordination compounds with such ligands have been reported in recent years (Mao *et al.*, 1999; Wu *et al.*, 2006; Zhang *et al.*, 2002).

The molecular structure is shown in Fig. 1. The Zn^II^ ion, lying on a twofold rotation axis, is coordinated by two Cl atoms and the two zwitterionic ligands through carboxylate O atoms, with a distorted tetrahedral coordination geometry. The Zn—Cl and Zn—O distances lie in the usual range for a tetrahedral Zn^II^ center (Table 1). The zwitterionic ligand is neutral with an anionic *N*-acetate group in a monodentate coordination mode and a cationic pyridinium ring. The acrylic group remains protonated and uncoordinated. This is in contrast with the only previous coordination compound with this ligand, [Ni_3_(C_10_H_8_NO_4_)_2_(N_3_)_4_(H_2_O)_2_].5H_2_O (Sun *et al.*, 2009), in which both carboxylate groups are deprotonated and coordinated in a bidentate bridging mode, giving an extended coordination network. As shown in Fig. 2, the mononuclear complex molecules in the title compound are associated into a one-dimensional chain along the [101] direction through intermolecular O—H···O hydrogen bonds (Table 2). The hydrogen bond involves the acrylic hydroxyl group (O4—H4B) from one molecule and the uncoordinated acetate O atom (O2) from another molecule.

### Experimental

The zwitterionic ligand was synthesized from ethyl pyridine-3-acrylate and ethyl bromoacetate according to the procedure for similar compounds (Mao *et al.*, 1999). A mixture of the ligand (0.021 g, 0.10 mmol), ZnCl_2_ (0.014 g, 0.10 mmol), NaN_3 _(0.026 g, 0.40 mmol), ethanol (1.0 ml) and H_2_O (2.0 ml) was sealed in a Teflon-lined autoclave and heated at 343 K for 3 d, then cooled to room temperature at a rate of 5 K h^-1^, giving a pale yellow solution. Slow evaporation of the solution at room temperature for several days afforded colorless block crystals of the title compound (yield 46%). Analysis, calculated for C_20_H_18_Cl_2_N_2_O_8_Zn: C 27.22, H 3.43, N 22.22%; found: C 27.11, H 3.82, N 22.50%. FT—IR (KBr, cm^-1^): 3530*m*, 3348br, 2084vs, 2059vs, 1630vs, 1582 s, 1505*m*, 1461w, 1388vs, 1307*m*, 985*m*.

### Refinement

H atoms attached to C atoms were placed at calculated positions and refined as riding atoms, with C—H = 0.93 (CH) and 0.97 (CH_2_) Å and with *U*_iso_(H) = 1.2*U*_eq_(C). The carboxylic H atom (H4B) was located from a difference Fourier map and refined isotropically.

### Crystal data

**Table:**

[ZnCl_2_(C_10_H_9_NO_4_)_2_]	*F*(000) = 1120
*M**_r_* = 550.63	*D*_x_ = 1.708 Mg m^−^^3^
Monoclinic, *C*2/*c*	Mo *K*α radiation, λ = 0.71073 Å
Hall symbol: -C 2yc	Cell parameters from 9202 reflections
*a* = 16.6247 (6) Å	θ = 2.3–26.0°
*b* = 7.1973 (3) Å	µ = 1.45 mm^−^^1^
*c* = 18.8873 (7) Å	*T* = 296 K
β = 108.685 (1)°	Block, colorless
*V* = 2140.81 (14) Å^3^	0.12 × 0.12 × 0.08 mm
*Z* = 4	

### Data collection

**Table:**

Bruker APEXII CCD diffractometer	2450 independent reflections
Radiation source: fine-focus sealed tube	2309 reflections with *I* > 2σ(*I*)
graphite	*R*_int_ = 0.019
φ and ω scans	θ_max_ = 27.5°, θ_min_ = 2.3°
Absorption correction: multi-scan (*SADABS*; Bruker, 2001)	*h* = −21→20
*T*_min_ = 0.845, *T*_max_ = 0.893	*k* = −9→8
27422 measured reflections	*l* = −24→23

### Refinement

**Table:**

Refinement on *F*^2^	Primary atom site location: structure-invariant direct methods
Least-squares matrix: full	Secondary atom site location: difference Fourier map
*R*[*F*^2^ > 2σ(*F*^2^)] = 0.028	Hydrogen site location: inferred from neighbouring sites
*wR*(*F*^2^) = 0.077	H atoms treated by a mixture of independent and constrained refinement
*S* = 1.06	*w* = 1/[σ^2^(*F*_o_^2^) + (0.0424*P*)^2^ + 2.553*P*] where *P* = (*F*_o_^2^ + 2*F*_c_^2^)/3
2450 reflections	(Δ/σ)_max_ < 0.001
154 parameters	Δρ_max_ = 0.50 e Å^−^^3^
0 restraints	Δρ_min_ = −0.23 e Å^−^^3^

### Fractional atomic coordinates and isotropic or equivalent isotropic displacement parameters (Å^2^)

**Table:**

	*x*	*y*	*z*	*U*_iso_*/*U*_eq_	
Zn1	0.0000	0.21438 (4)	0.2500	0.02879 (10)	
C1	0.10900 (11)	0.5110 (3)	0.25776 (10)	0.0324 (4)	
C2	0.14353 (12)	0.6811 (3)	0.22916 (10)	0.0360 (4)	
H2A	0.1139	0.7904	0.2379	0.043*	
H2B	0.2032	0.6953	0.2576	0.043*	
C3	0.20158 (11)	0.6192 (3)	0.12864 (11)	0.0370 (4)	
H3A	0.2525	0.5883	0.1649	0.044*	
C4	0.19519 (13)	0.6114 (3)	0.05458 (12)	0.0432 (4)	
H4A	0.2413	0.5734	0.0405	0.052*	
C5	0.12009 (13)	0.6600 (3)	0.00115 (11)	0.0395 (4)	
H5A	0.1155	0.6563	−0.0492	0.047*	
C6	0.06027 (11)	0.7173 (2)	0.09822 (10)	0.0318 (4)	
H6A	0.0146	0.7511	0.1137	0.038*	
C7	0.05107 (12)	0.7146 (2)	0.02242 (10)	0.0323 (4)	
C8	−0.02829 (13)	0.7712 (3)	−0.03508 (10)	0.0371 (4)	
H8A	−0.0273	0.7784	−0.0840	0.044*	
C9	−0.10021 (13)	0.8126 (3)	−0.02414 (11)	0.0401 (4)	
H9A	−0.1045	0.8061	0.0237	0.048*	
C10	−0.17521 (12)	0.8700 (3)	−0.08844 (10)	0.0380 (4)	
N1	0.13464 (9)	0.6713 (2)	0.14914 (8)	0.0290 (3)	
O1	0.06462 (9)	0.39902 (19)	0.21043 (7)	0.0371 (3)	
O2	0.12548 (10)	0.5002 (2)	0.32652 (7)	0.0509 (4)	
O3	−0.17174 (10)	0.9091 (3)	−0.14938 (9)	0.0574 (4)	
O4	−0.24415 (10)	0.8769 (3)	−0.07066 (9)	0.0592 (5)	
H4B	−0.290 (2)	0.919 (5)	−0.1137 (19)	0.091 (11)*	
Cl1	0.07978 (3)	0.02552 (6)	0.34083 (2)	0.03619 (12)	

### Atomic displacement parameters (Å^2^)

**Table:**

	*U*^11^	*U*^22^	*U*^33^	*U*^12^	*U*^13^	*U*^23^
Zn1	0.03138 (16)	0.03059 (16)	0.02483 (15)	0.000	0.00960 (11)	0.000
C1	0.0285 (8)	0.0387 (9)	0.0288 (8)	−0.0027 (7)	0.0075 (6)	0.0018 (7)
C2	0.0417 (9)	0.0378 (9)	0.0263 (8)	−0.0084 (8)	0.0076 (7)	−0.0012 (7)
C3	0.0288 (8)	0.0389 (9)	0.0422 (9)	0.0025 (7)	0.0099 (7)	0.0063 (8)
C4	0.0391 (10)	0.0484 (11)	0.0485 (11)	0.0031 (8)	0.0231 (8)	0.0044 (9)
C5	0.0492 (11)	0.0384 (9)	0.0343 (9)	−0.0016 (8)	0.0183 (8)	0.0024 (8)
C6	0.0289 (8)	0.0313 (8)	0.0345 (9)	0.0009 (6)	0.0090 (7)	0.0001 (7)
C7	0.0356 (9)	0.0271 (8)	0.0308 (8)	−0.0020 (6)	0.0059 (7)	0.0030 (6)
C8	0.0411 (10)	0.0382 (9)	0.0283 (8)	−0.0014 (8)	0.0060 (7)	0.0027 (7)
C9	0.0447 (10)	0.0434 (10)	0.0292 (9)	0.0027 (8)	0.0077 (8)	−0.0009 (8)
C10	0.0422 (10)	0.0332 (9)	0.0329 (9)	0.0023 (7)	0.0040 (7)	−0.0018 (7)
N1	0.0304 (7)	0.0282 (6)	0.0268 (7)	−0.0021 (5)	0.0069 (5)	0.0026 (5)
O1	0.0436 (7)	0.0394 (7)	0.0300 (6)	−0.0126 (6)	0.0142 (5)	−0.0051 (5)
O2	0.0545 (9)	0.0663 (10)	0.0266 (6)	−0.0263 (8)	0.0056 (6)	0.0030 (6)
O3	0.0447 (8)	0.0801 (12)	0.0441 (8)	0.0034 (8)	0.0094 (7)	0.0092 (8)
O4	0.0445 (8)	0.0929 (14)	0.0360 (8)	0.0210 (9)	0.0070 (6)	0.0123 (8)
Cl1	0.0369 (2)	0.0387 (2)	0.0321 (2)	0.00658 (17)	0.00991 (17)	0.00704 (16)

### Geometric parameters (Å, °)

**Table:**

Zn1—O1	1.9983 (13)	C5—C7	1.388 (3)
Zn1—Cl1	2.2566 (4)	C5—H5A	0.9300
C1—O2	1.241 (2)	C6—N1	1.342 (2)
C1—O1	1.253 (2)	C6—C7	1.390 (3)
C1—C2	1.522 (3)	C6—H6A	0.9300
C2—N1	1.472 (2)	C7—C8	1.473 (2)
C2—H2A	0.9700	C8—C9	1.311 (3)
C2—H2B	0.9700	C8—H8A	0.9300
C3—N1	1.343 (2)	C9—C10	1.493 (3)
C3—C4	1.370 (3)	C9—H9A	0.9300
C3—H3A	0.9300	C10—O3	1.204 (3)
C4—C5	1.376 (3)	C10—O4	1.294 (3)
C4—H4A	0.9300	O4—H4B	0.97 (4)
			
O1^i^—Zn1—O1	96.63 (8)	C4—C5—H5A	120.0
O1^i^—Zn1—Cl1^i^	115.43 (4)	C7—C5—H5A	120.0
O1—Zn1—Cl1^i^	111.82 (4)	N1—C6—C7	120.43 (17)
O1^i^—Zn1—Cl1	111.82 (4)	N1—C6—H6A	119.8
O1—Zn1—Cl1	115.43 (4)	C7—C6—H6A	119.8
Cl1^i^—Zn1—Cl1	105.92 (3)	C5—C7—C6	118.26 (17)
O2—C1—O1	126.04 (17)	C5—C7—C8	119.55 (17)
O2—C1—C2	116.10 (16)	C6—C7—C8	122.18 (17)
O1—C1—C2	117.82 (15)	C9—C8—C7	126.40 (18)
N1—C2—C1	113.62 (15)	C9—C8—H8A	116.8
N1—C2—H2A	108.8	C7—C8—H8A	116.8
C1—C2—H2A	108.8	C8—C9—C10	120.04 (18)
N1—C2—H2B	108.8	C8—C9—H9A	120.0
C1—C2—H2B	108.8	C10—C9—H9A	120.0
H2A—C2—H2B	107.7	O3—C10—O4	123.81 (18)
N1—C3—C4	120.36 (17)	O3—C10—C9	123.98 (19)
N1—C3—H3A	119.8	O4—C10—C9	112.19 (17)
C4—C3—H3A	119.8	C6—N1—C3	121.35 (16)
C3—C4—C5	119.60 (18)	C6—N1—C2	119.36 (15)
C3—C4—H4A	120.2	C3—N1—C2	119.28 (15)
C5—C4—H4A	120.2	C1—O1—Zn1	115.16 (11)
C4—C5—C7	119.98 (18)	C10—O4—H4B	109 (2)
			
O2—C1—C2—N1	170.27 (17)	C8—C9—C10—O4	169.7 (2)
O1—C1—C2—N1	−12.1 (2)	C7—C6—N1—C3	−1.0 (3)
N1—C3—C4—C5	1.0 (3)	C7—C6—N1—C2	178.06 (16)
C3—C4—C5—C7	−0.7 (3)	C4—C3—N1—C6	−0.2 (3)
C4—C5—C7—C6	−0.4 (3)	C4—C3—N1—C2	−179.18 (18)
C4—C5—C7—C8	178.33 (19)	C1—C2—N1—C6	82.1 (2)
N1—C6—C7—C5	1.2 (3)	C1—C2—N1—C3	−98.9 (2)
N1—C6—C7—C8	−177.45 (16)	O2—C1—O1—Zn1	11.0 (3)
C5—C7—C8—C9	174.5 (2)	C2—C1—O1—Zn1	−166.35 (13)
C6—C7—C8—C9	−6.8 (3)	O1^i^—Zn1—O1—C1	57.77 (12)
C7—C8—C9—C10	179.31 (18)	Cl1^i^—Zn1—O1—C1	178.59 (12)
C8—C9—C10—O3	−11.8 (3)	Cl1—Zn1—O1—C1	−60.24 (14)

Symmetry codes: (i) −*x*, *y*, −*z*+1/2.

### Hydrogen-bond geometry (Å, °)

**Table:**

*D*—H···*A*	*D*—H	H···*A*	*D*···*A*	*D*—H···*A*
O4—H4B···O2^ii^	0.97 (4)	1.60 (4)	2.560 (2)	169 (3)

Symmetry codes: (ii) *x*−1/2, −*y*+3/2, *z*−1/2.
